# Global Trends and Research Hot Spots in Medication Regimen Simplification: Bibliometric Analysis

**DOI:** 10.2196/82274

**Published:** 2026-05-29

**Authors:** Chuwen Dou, Bei Wu, Leyi Wu, An Gu, An Huang, Chen Zhang, Xichenhui Qiu, Jianlin Lou, Lina Wang

**Affiliations:** 1 Huzhou Key Laboratory of Precise Prevention and Control of Major Chronic Diseases, School of Medicine, Huzhou Normal University, No. 168 Wuxing Road, Wuxing District, Huzhou, Zhejiang, 313000, China, 86 13587278357; 2 Faculty of Arts and Science, New York University Shanghai, Shanghai, China; 3 Department of General Medicine, Community Health Service Center of Renhuangshan, Huzhou, Huzhou, China; 4 Health Science Center, Shenzhen University, Shenzhen, China

**Keywords:** medication regimen simplification, bibliometric analysis, polypharmacy, medication adherence, medication regimen complexity

## Abstract

**Background:**

Medication regimen simplification has gained increasing attention as a strategy to reduce treatment burden and improve medication use. However, the overall development, knowledge structure, and emerging themes of this field have not been systematically mapped, hindering efforts to identify clear research priorities and support strategies that facilitate the translation of simplified approaches into optimized medication management.

**Objective:**

This study aimed to examine global research trends, collaboration patterns, and research hot spots in medication regimen simplification.

**Methods:**

A bibliometric analysis was conducted to characterize the research landscape of medication regimen simplification. Publications from the Web of Science Core Collection (January 1, 2005, to December 31, 2025) were analyzed using Microsoft Excel 365, VOSviewer, CiteSpace, and Bibliometrix in R for publication trends, countries and regions, institutions, authors, cocited references, keyword co-occurrence, clustering, and thematic evolution.

**Results:**

A total of 468 publications were included in this bibliometric analysis. Annual publication output showed an upward trend, although fluctuations occurred across years. The United States ranked first in publication output and total citations, and Monash University was the most productive institution. Collaboration was concentrated in a limited number of country, institutional, and author clusters, with the strongest international links between North America and Europe. Cocitation and keyword analyses showed that the field evolved from a focus on HIV-related regimen optimization and medication adherence to broader themes in chronic disease management, including type 2 diabetes, heart failure, long-term care, and medication regimen complexity.

**Conclusions:**

Research on medication regimen simplification has grown over time, but collaboration remains relatively concentrated. Current hot spots center on its use in specific disease settings and on medication complexity and adherence, whereas recent trends highlight real-world implementation. Future research should further examine the long-term impact and sustainability of simplification strategies.

## Introduction

With advances in medical care, more patients with chronic conditions such as hypertension, diabetes, and cardiovascular disease require multiple medications for long-term pharmacotherapy. However, the benefits of treatment are frequently limited by poor medication adherence, and complex drug regimens are an important contributing factor [1,2]. More complex medication regimens are associated with poorer adherence, which may compromise disease control and increase the risk of adverse outcomes [3]. Older adults, particularly those with cognitive impairment or limited health literacy, often experience difficulties with medication management, which may contribute to unintentional nonadherence, medication errors, and reduced quality of life [4-6].

Complex medication regimens in older adults, particularly those involving multiple drugs and frequent dosing, can make adherence more difficult [2]. Polypharmacy may also increase the risk of drug-drug interactions and health care costs [7]. As a result, medication regimen simplification has become a key strategy to optimize pharmacotherapy, improve adherence, and reduce treatment burden [8,9]. Its importance is further underscored by the World Health Organization’s advocacy for the rational use of medicines, ensuring that patients receive medications appropriate to their clinical needs and in correct doses for effective outcomes [10]. Similarly, a Centers for Disease Control and Prevention Grand Rounds report highlighted improved medication adherence as a key component of chronic disease management and optimal prescribing practices [11]. In clinical practice, regimen simplification may be considered as part of broader medication review and optimization processes [12].

Medication regimen simplification refers to reducing the complexity of medication use through strategies such as consolidating dosing times, reducing dosing frequency, standardizing administration routes, substituting long-acting for short-acting formulations, and using fixed-dose or other combination products when possible without altering the therapeutic goals, efficacy, or safety of treatment [8]. Importantly, medication regimen complexity is not determined solely by the number of medicines prescribed but also by dosage forms, dosing frequency, administration times, routes of administration, and additional instructions required for proper use [13-15]. Accordingly, medication regimen simplification aims to reduce the practical and cognitive burden of medication use for patients or caregivers [8]. Its application spans a wide range of clinical contexts, including polypharmacy in older adults; chronic disease management; HIV care; and other settings characterized by high regimen complexity, such as long-term care, home-based care, and multi-morbidity [16,17]. This concept is distinct from deprescribing, which primarily involves discontinuing inappropriate or unnecessary medications, whereas medication regimen simplification seeks to make an otherwise appropriate regimen easier to follow without changing its therapeutic intent [16,18]. It should also be distinguished from broader concepts such as medication optimization, medication review, rational prescribing, and adherence-enhancing interventions [19-21]. In this sense, simplification may be regarded as one component of medication optimization and one possible outcome of medication review, but it is not synonymous with either. In this study, reductions in medication regimen complexity index scores or simplification of medication administration processes were considered relevant only when they reflected a deliberate effort to simplify the medication regimen actually used by patients or caregivers [22,23].

Despite growing interest in this area, related research remains dispersed across populations, disease settings, and intervention types, and the overall intellectual structure and development of the field have not been systematically characterized. Bibliometric analysis offers a systematic and visual approach to map the structure, trends, and knowledge gaps of a research field [24,25]. This study used VOSviewer (Centre for Science and Technology Studies, Leiden University) and CiteSpace to analyze the global literature on medication regimen simplification and identify key contributors, major research themes, and emerging trends with the aim of informing future research and clinical practice.

## Methods

### Search Strategy and Eligibility Criteria

Publications were retrieved from the Web of Science Core Collection (WoSCC). The search was conducted on March 6, 2026, and covered the period from January 1, 2005, to December 31, 2025. All records were obtained from the Science Citation Index Expanded within WoSCC. The search was performed in the title, author keywords, and abstract fields. Only articles and reviews published in English were included. Because “medication regimen simplification” is neither a standardized indexing term nor a MeSH (Medical Subject Headings) term, the search strategy relied on a combination of free-text terms informed by related expressions reported in previous studies and by the conceptual scope of this study [8,9,26]. These terms included simplification-related expressions for regimens, medications, and therapies, as well as expressions related to reduced medication regimen complexity. The full search strategy is shown in Table 1.

**Table 1.** Summary of data source and study selection.

Item	Description
Data source	WoSCC^a^
Search date	March 6, 2026
Time span	2005-2025
Language	English
Document type	Articles and reviews
Search field	Title, author keywords, and abstract
Search strategy	TI/AK/AB = (“regimen simplification” OR “treatment simplification” OR “therapy simplification” OR “medication regimen simplification” OR “simplified regimen” OR “simplified medication regimen” OR (simplif* NEAR/3 regimen*) OR (simplif* NEAR/3 medication*) OR (simplif* NEAR/3 therap*) OR (“medication regimen complexity” NEAR/3 reduc*) OR (reduce* NEAR/3 “medication regimen complexity”))
Initial records retrieved, n	1466
Screening criteria	Records were screened by title and abstract. Publications were excluded if they did not describe medication regimen simplification as a primary focus or if any reported reduction in medication regimen complexity was not presented as an intended simplification strategy.
Final records included, n	468

a WoSCC: Web of Science Core Collection.

In this study, medication regimen simplification was operationally defined as strategies intended to reduce the complexity of a medication regimen while preserving its therapeutic intent. On the basis of this definition, this bibliometric analysis included publications that explicitly described such strategies as medication regimen simplification or clearly characterized them as a reduction in medication regimen complexity. Interventions such as reduced dosing frequency, consolidated administration times, long-acting formulations, and fixed-dose combinations were included only when they were presented as intended to simplify the regimen or reduce its complexity. Deprescribing was not considered equivalent to medication regimen simplification. Similarly, lower medication regimen complexity scores or simpler medication administration schedules were considered relevant only when they reflected an explicit strategy to simplify the regimen followed by patients or caregivers.

### Deduplication and Screening Reliability

Two researchers independently screened the titles and abstracts of all retrieved records. Full texts of potentially eligible studies were then reviewed independently by the same 2 researchers according to the predefined inclusion and exclusion criteria. Any disagreements were resolved through discussion with a third researcher. Manual checking was performed to identify any remaining duplicate records or overlapping reports of the same study. In addition, the reference lists of included studies were screened manually to identify potentially relevant articles. Interrater agreement between the 2 researchers was assessed using the Cohen κ, which indicated a high level of agreement (κ=0.83).

### Data Extraction and Collection

Bibliometric data were extracted from the included publications, including publication year, country or region, institution, author, journal, keywords, cited references, and research area information, as well as annual publication counts. Before analysis, limited manual corrections were performed to improve the consistency of country, institution, and author information. Institution names were merged when spelling variants, abbreviations, or formatting differences referred to the same organization. Country names were standardized when the WoSCC classification was evidently inconsistent. Author names were standardized only for obvious variants referring to the same individual based on publication context and affiliation information. In addition, the names of the top authors and institutions reported in the main tables were manually cross-checked against the original WoSCC records and affiliation information.

### Data Processing

CiteSpace (version 6.4.R1 Advanced) [27] and VOSviewer (version 1.6.20) [28] were used for bibliometric visualization and network analysis. CiteSpace was used to analyze the dual-map overlay of journals, reference cocitation, keyword clustering, and burst detection. VOSviewer was used to visualize collaboration networks among countries and regions, institutions, and authors, as well as keyword co-occurrence. In addition, Bibliometrix in R (version 4.4.1; K-Synth Srl) [29] was used for supplementary visualization of countries and regions and keyword data, and Microsoft Excel 365 was used to present annual publication trends.

For network construction in VOSviewer, the following thresholds were applied: keywords with at least 3 occurrences, countries and regions with at least 5 publications, institutions with at least 3 publications, and authors with at least 2 publications were included. These thresholds were chosen to keep the networks interpretable and reduce noise from low-frequency items [30]. In CiteSpace, the time span was set from 2005 to 2025 with 1 year per slice, and Pathfinder pruning was applied to simplify the network structure and highlight the most meaningful links. Betweenness centrality was calculated by CiteSpace using its default settings and was used to identify important nodes in the networks.

### Ethical Considerations

This study was based on publicly available literature and did not involve human or animal participants; thus, no ethics approval was required.

## Results

### Study Selection

**Figure 1.**
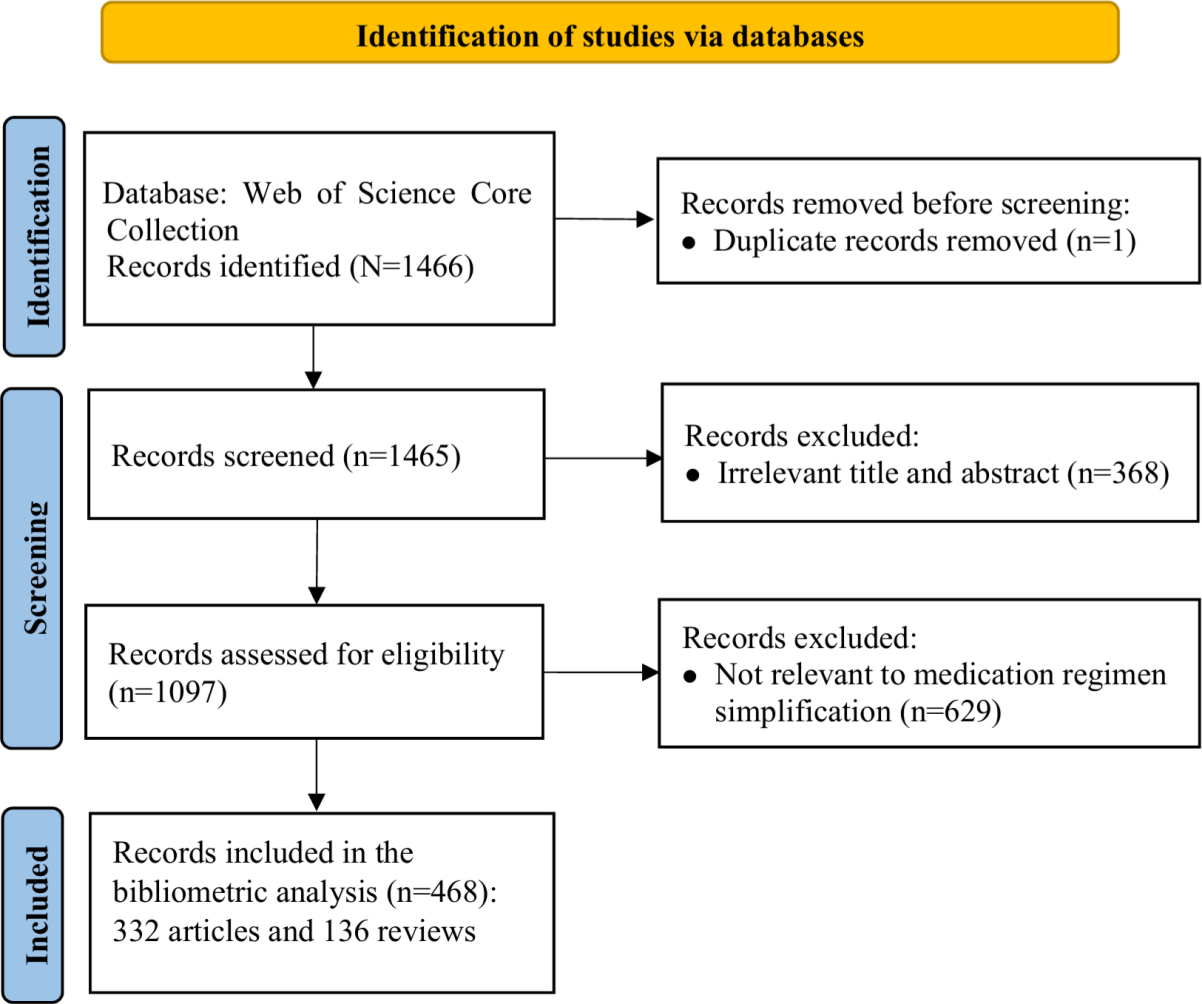
Flowchart of the study selection process.

A total of 1466 records were identified. After title and abstract screening, publications without an explicit focus on medication regimen simplification or reduction in medication regimen complexity were excluded. Ultimately, of the 1466 records, 468 (31.9%) publications were included in the final analysis. The study selection process is shown in Figure 1.

### Annual Publication Statistics

**Figure 2.**
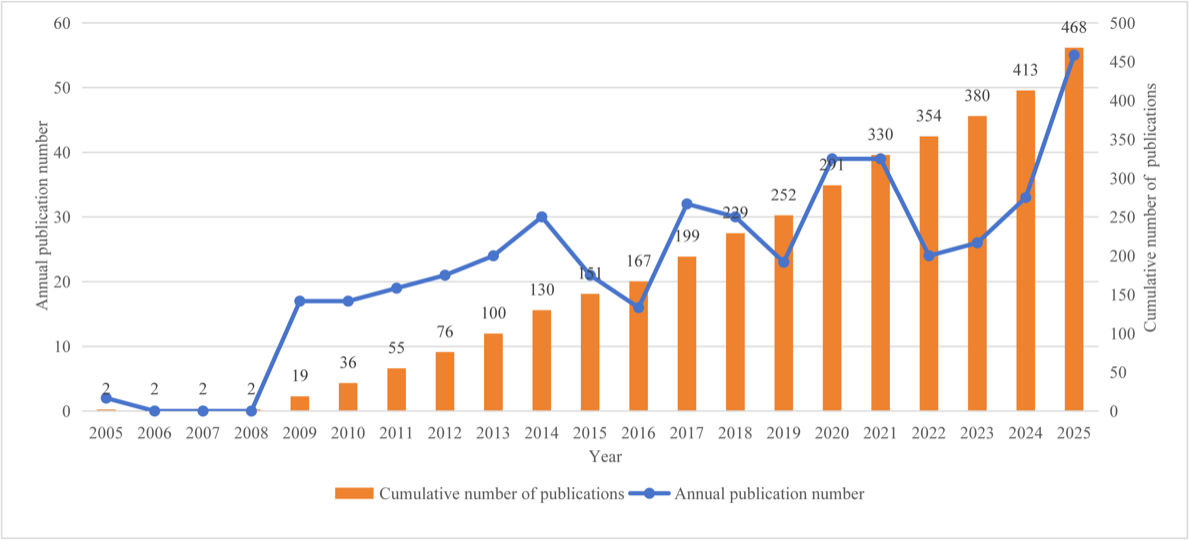
Annual volume of publications.

Figure 2 illustrates the annual and cumulative number of publications on medication regimen simplification from 2005 to 2025. Annual publication output was low from 2005 to 2008 and then increased from 2009 onward, with fluctuations across years. The cumulative number of publications increased throughout the study period, from 2 in 2005 to 468 in 2025.

### Global Contribution of Countries and Regions, Institutions, and Authors to the Field

#### Countries and Regions

**Figure 3.**
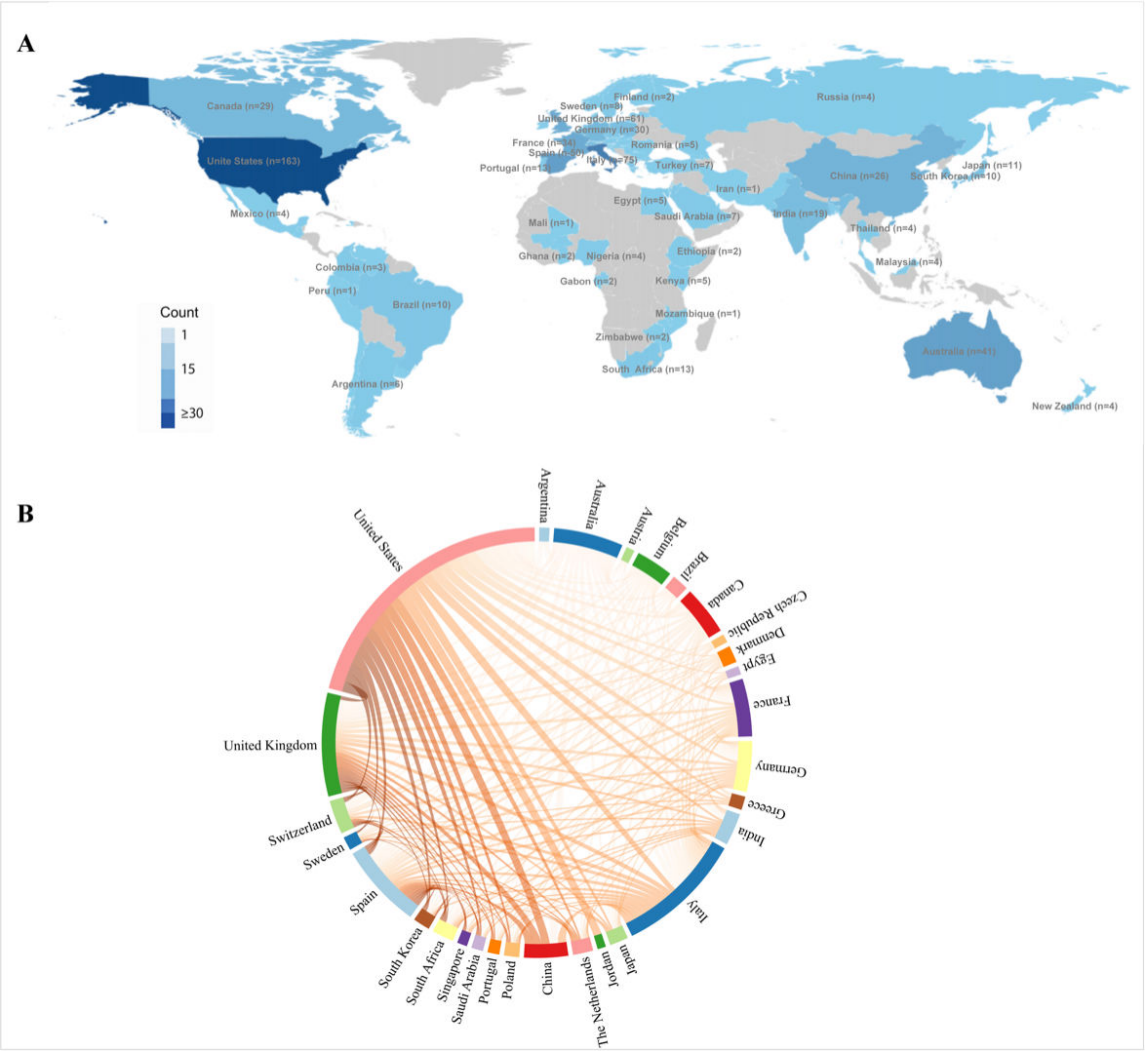
(A) Global distribution of publications (2005-2025) and (B) networks of country cooperation. UK: United Kingdom.

Figure 3A shows the distribution of publications on medication regimen simplification across countries and regions from 2005 to 2025. The 10 most productive countries and regions and their citation counts are listed in Multimedia Appendix 1. The United States contributed the largest number of publications (163/468, 34.8%), followed by Italy (75/468, 16.0%) and the United Kingdom (61/468, 13.0%). The United States also had the highest citation count (n=6720), followed by Italy (n=3800). Figure 3B shows the country and region collaboration network, in which the United States, the United Kingdom, Italy, Germany, China, and Australia had larger nodes and multiple links with other countries and regions. Cross-national links were most evident between North America and Europe, with additional links involving East Asia and Oceania.

#### Institutions

**Figure 4.**
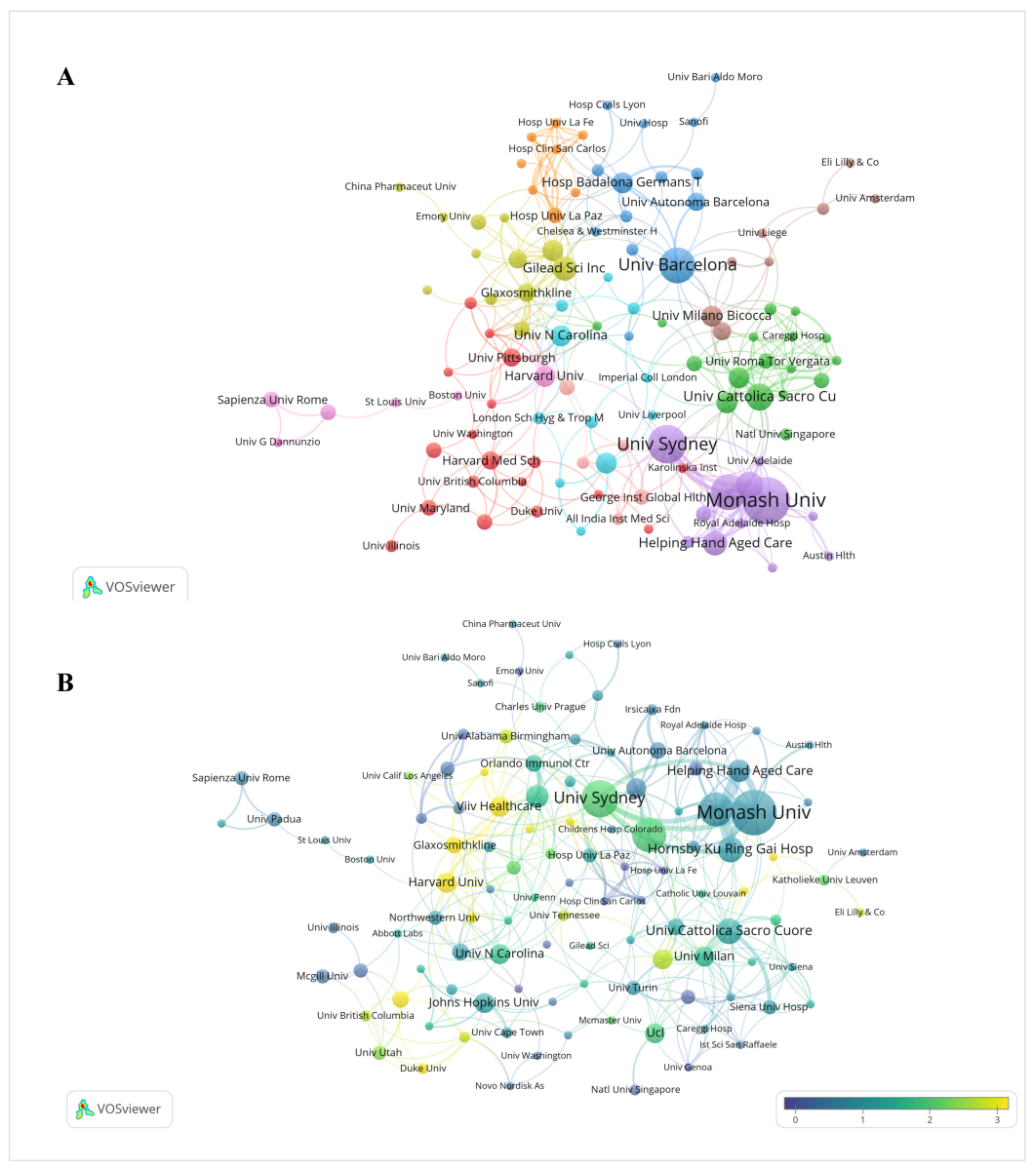
(A) Institutional cooperation network and (B) overlay visualization of the institution network based on average normalized citations.

Table 2 presents the 10 institutions with the highest publication counts and total citations. A total of 2234 institutions were identified. Monash University had the highest number of publications (16/468, 3.4%), followed by the University of Sydney (13/468, 2.8%). Among the top 10 most productive institutions, 5 (50%) were from Australia, 2 (20%) were from the United States, and 2 (20%) were from Spain. The University of Sydney had the highest total citation count (n=1764), followed by Harvard University (n=1740) and the University of Barcelona (n=550). Figure 4A shows the institutional collaboration network, in which multiple clusters are visible. Figure 4B presents the overlay visualization based on average normalized citations. Harvard University, Duke University, and Vivli are located in relatively warmer areas of the network, indicating higher average normalized citations.

**Table 2.** Top 10 institutions by publication count and total citations.

Rank	Institution	Country	Studies (n=468), n (%)	Total citations, n
1	Monash University	Australia	16 (3.4)	333
2	University of Sydney	Australia	13 (2.8)	1764
3	University of Barcelona	Spain	12 (2.6)	550
4	University of South Australia	Australia	12 (2.6)	220
5	Hornsby Ku-ring-gai Hospital	Australia	9 (1.9)	213
6	Catholic University of the Sacred Heart	Italy	9 (1.9)	329
7	Gilead Sciences	United States	8 (1.7)	440
8	Helping Hand	Australia	8 (1.7)	149
9	Harvard University	United States	7 (1.5)	1740
10	Hospital Germans Trias i Pujol	Spain	7 (1.5)	125

#### Authors

**Figure 5.**
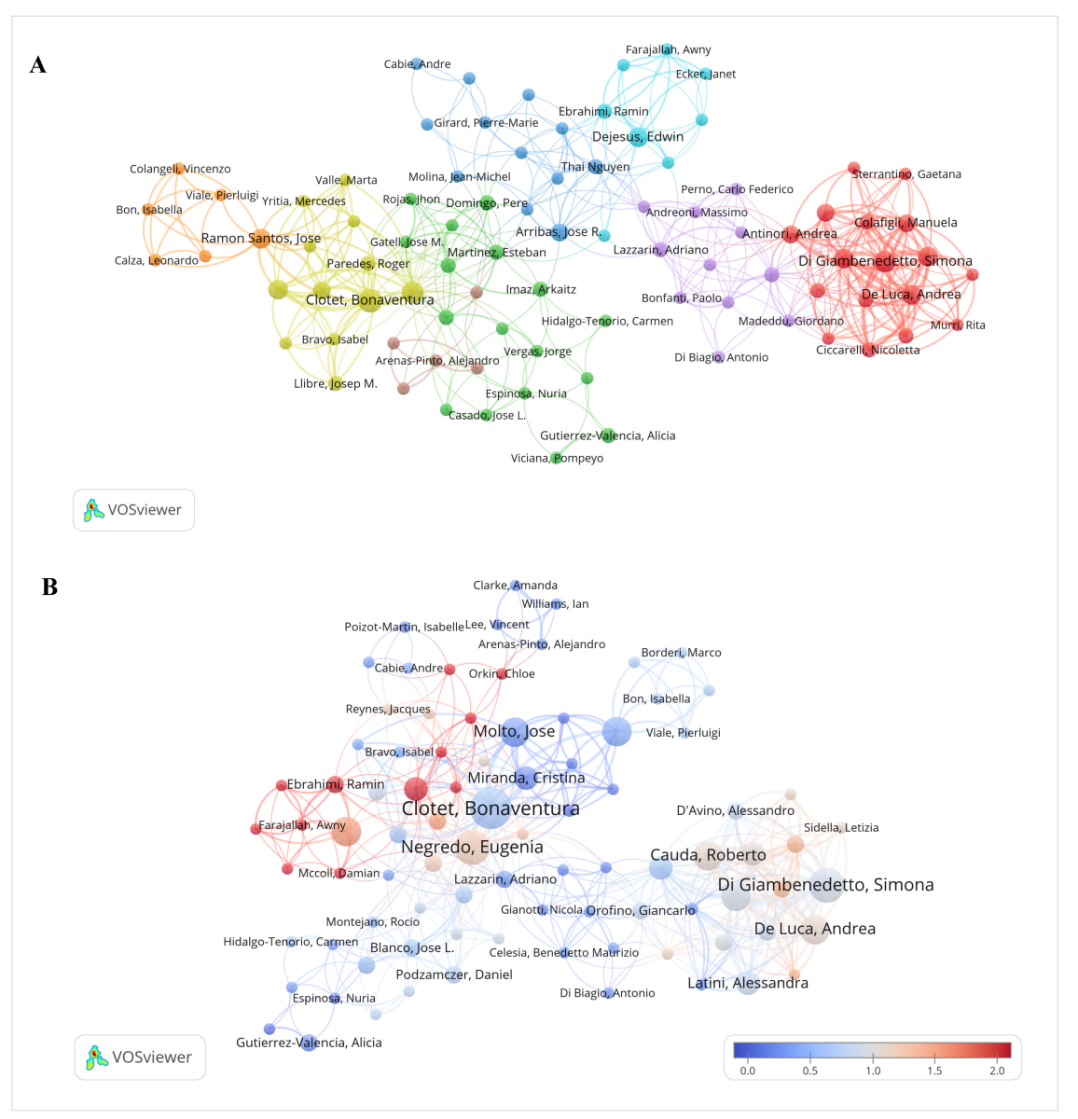
(A) Network map of author cooperation (2005-2025) and (B) overlay visualization of the author network based on average normalized citations.

A total of 3109 authors contributed to the 468 publications on medication regimen simplification. Figure 5A shows the author collaboration network, in which multiple clusters are visible. Clotet, Di Giambenedetto, and De Luca are located in connected parts of the network and are linked with multiple coauthors. Figure 5B shows the overlay visualization of the author network based on average normalized citations. Warmer colors indicate higher citation impact. Clotet, Ebrahim Ramin, Farajzadeh Awny, and Di Giambenedetto are represented by relatively warmer-colored nodes. Table 3 shows author-level metrics. Bell had the highest number of publications (12/468, 2.6%), followed by Sluggett (11/468, 2.4%), and both authors had an H-index of 9. Di Giambenedetto had the highest total citation count (n=260), followed by De Luca (n=254), Bell (n=239), and Sluggett (n=237).

**Table 3.** Most prolific authors based on author-level metrics.

Author	Publications (n=468), n (%)	H-index^a^	TC^b^, n	PY_start^c^
Bell	12 (2.6)	9	239	2018
Sluggett	11 (2.4)	9	237	2018
Chen	8 (1.7)	8	155	2018
Corlis	9 (1.9)	8	150	2018
De Luca	7 (1.5)	7	254	2013
Di Giambenedetto	8 (1.7)	7	260	2013
Van Emden	7 (1.5)	7	121	2018
Caporale	6 (1.3)	6	112	2018
Cauda	6 (1.3)	6	217	2013
Clotet	7 (1.5)	6	132	2009

a H-index: Hirsch index; h publications cited at least h times each.

b TC: total citations.

c PY_start: author’s first publication year in the dataset.

### Reference Cocitation and Keyword Burst Analysis

**Figure 6.**
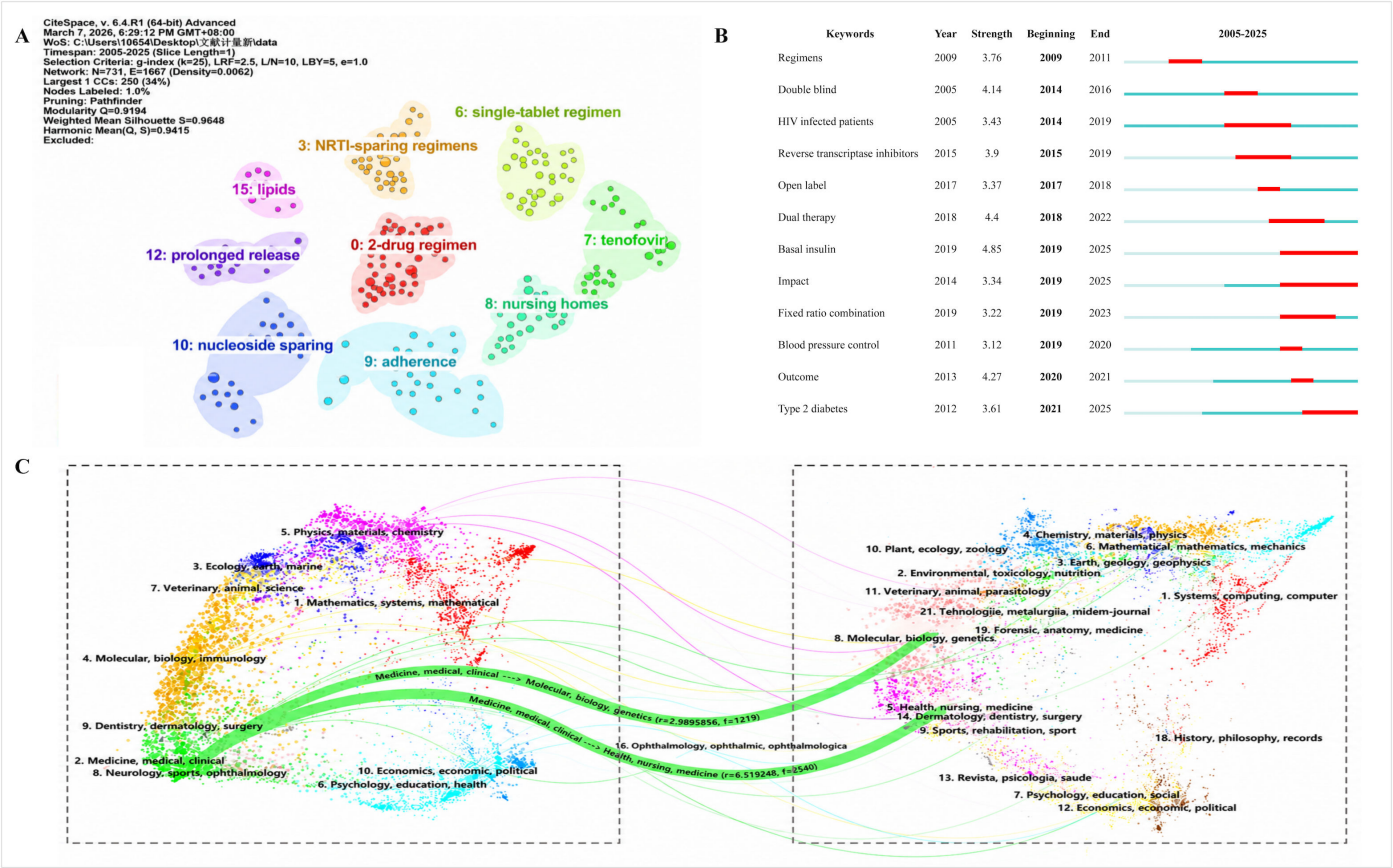
(A) Reference cocitation clusters, (B) top 12 keywords with the strongest citation bursts (2005-2025), and (C) the dual-map overlay of journals publishing studies on medication regimen simplification from 2005 to 2025 (left: citing journals; right: cited journals). NRTI: nucleoside or nucleotide reverse transcriptase inhibitors.

Figure 6A shows the reference cocitation clusters. Using CiteSpace with a 1-year time slice from 2005 to 2025, the network included 731 nodes and 1667 links, with a modularity *Q* of 0.919 and a silhouette score of 0.964. The main labeled clusters included 2-drug regimen (cluster 0), nucleoside or nucleotide reverse transcriptase inhibitors–sparing regimens (cluster 3), single-tablet regimen (cluster 6), tenofovir (cluster 7), nursing homes (cluster 8), and adherence (cluster 9). Figure 6B shows the 12 keywords with the strongest citation bursts. Burst keywords occurring earlier in the study period included “regimens,” “double blind,” “HIV infected patients,” “reverse transcriptase inhibitors,” and “open label,” whereas burst keywords extending into later years included “dual therapy,” “basal insulin,” “fixed ratio combination,” “blood pressure control,” “impact,” and “type 2 diabetes.” The burst keywords “dual therapy” and “regimens” overlapped with the regimen-related cocitation clusters in Figure 6A, including 2-drug regimen (cluster 0), nucleoside or nucleotide reverse transcriptase inhibitors–sparing regimens (cluster 3), and single-tablet regimen (cluster 6).

### Changing Trends in Research Disciplines

As shown in Figure 6C, the dual-map overlay reveals the knowledge flow between disciplines in the field of medication regimen simplification. The main citation paths originated from journals in the medicine, medical, and clinical area and extended to journals in the molecular, biology, and genetics area and the health, nursing, and medicine area. The most prominent paths are shown in green.

### Keyword Analysis

#### Keyword Co-Occurrence

**Figure 7.**
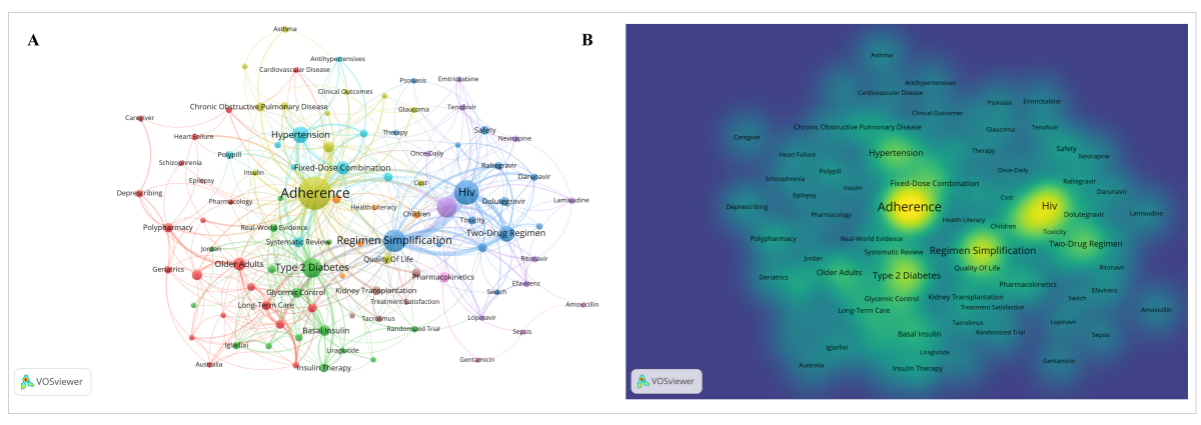
(A) Keyword co-occurrence network and (B) density visualization of keyword co-occurrence.

Figure 7A shows the keyword co-occurrence network generated using VOSviewer. Using all keywords as the unit of analysis, and after removal of irrelevant terms, 100 keywords with at least 3 occurrences were included. The most frequent keywords were “efficacy” (n=63), “therapy” (n=61), and “adherence” (n=61; Multimedia Appendix 2). In Figure 7A, “adherence,” “HIV,” “regimen simplification,” “type 2 diabetes,” and “fixed-dose combination” are located in connected parts of the network and have multiple links with other keywords. Figure 7B shows the density visualization of keyword co-occurrence. Areas of higher density are visible around “adherence,” “HIV,” and “regimen simplification,” with additional dense areas around “type 2 diabetes” and “fixed-dose combination.”

#### Keyword Cluster Analysis

**Figure 8.**
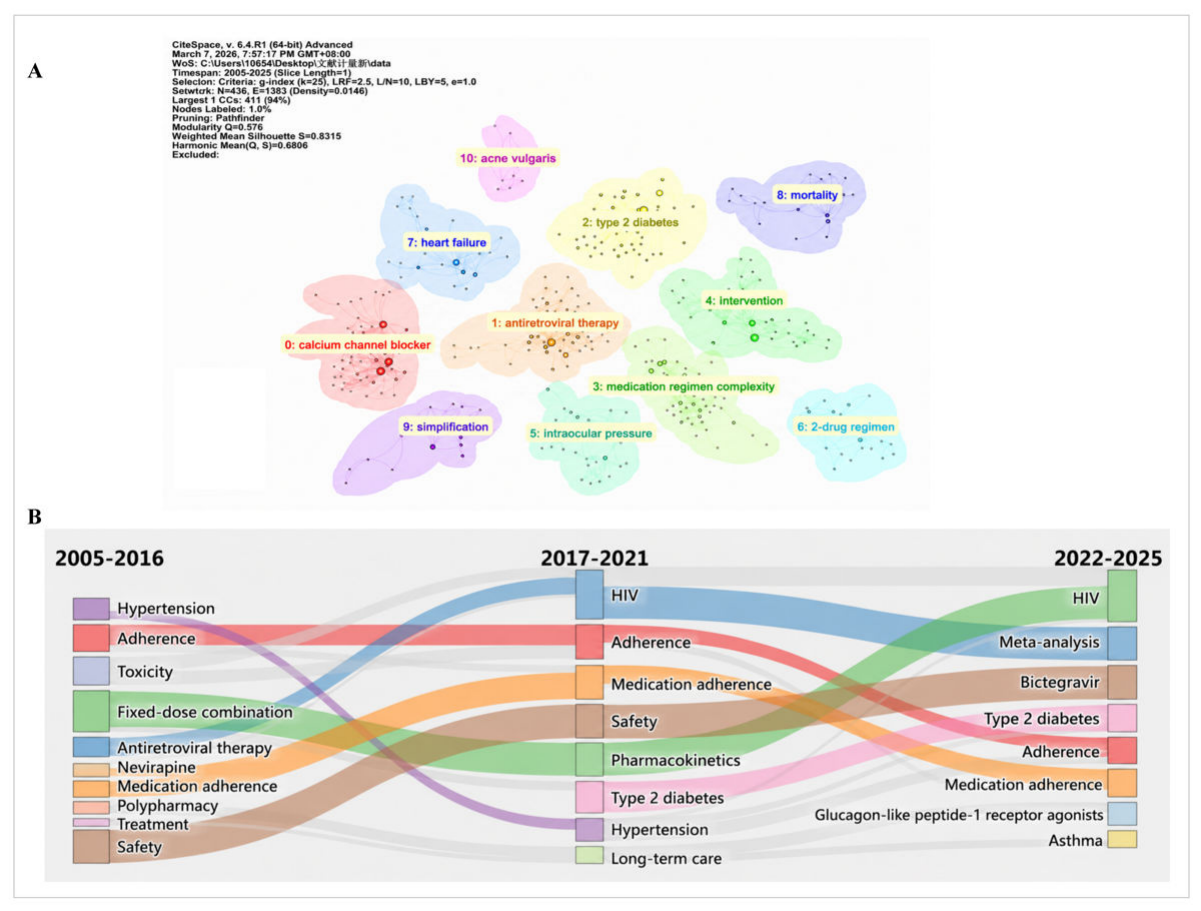
(A) Keyword clustering map and (B) thematic evolution of keywords (2005‐2025).

Figure 8A shows the keyword clustering network generated by CiteSpace. Using the log-likelihood ratio algorithm, 11 clusters were identified. The network had a modularity *Q* of 0.576 and silhouette score of 0.831. The labeled clusters were calcium channel blocker (cluster 0), antiretroviral therapy (cluster 1), type 2 diabetes (cluster 2), medication regimen complexity (cluster 3), intervention (cluster 4), intraocular pressure (cluster 5), 2-drug regimen (cluster 6), heart failure (cluster 7), mortality (cluster 8), simplification (cluster 9), and acne vulgaris (cluster 10).

### Thematic Evolution

Figure 8B shows the thematic evolution of keywords across 3 periods: 2005 to 2016, 2017 to 2021, and 2022 to 2025. From 2005 to 2016, the main keywords included “hypertension,” “adherence,” “toxicity,” “fixed-dose combination,” “antiretroviral therapy,” “nevirapine,” “medication adherence,” “polypharmacy,” “treatment,” and “safety.” From 2017 to 2021, the main keywords included “HIV,” “adherence,” “medication adherence,” “safety,” “pharmacokinetics,” “type 2 diabetes,” “hypertension,” and “long-term care.” From 2022 to 2025, the main keywords included “HIV,” “meta-analysis,” “bictegravir,” “type 2 diabetes,” “adherence,” “medication adherence,” “glucagon-like peptide-1 receptor agonists,” and “asthma.” Links between periods are shown for several keywords, including “adherence,” “medication adherence,” “HIV,” “type 2 diabetes,” and “safety.”

## Discussion

### Principal Findings

This study provides a bibliometric overview of research on medication regimen simplification from 2005 to 2025. The findings show increasing publication output over time, with research activity concentrated in a limited number of countries, institutions, and author groups, whereas the intellectual structure and evolving keyword patterns indicate a field centered on regimen simplification, adherence, and disease-specific applications.

At the country level, research on medication regimen simplification was dominated by a limited number of contributors. The United States ranked first in both publication output and total citations, possibly reflecting not only the size of its research community and funding environment [31,32] but also broader factors that influence bibliometric visibility, including English-language publication and database coverage. At the institutional level, Monash University and the University of Sydney were among the leading contributors, and 50% (5/10) of the most productive institutions were from Australia. Monash University’s prominent contribution may reflect both its sustained research on medication use, medication safety, healthy aging, and aged care [33] and the broader Australian policy and practice focus on polypharmacy and medication management in older adults [34,35]. Notably, several productive institutions were hospitals, aged care providers, or industry-affiliated institutions, suggesting that medication regimen simplification is often closely linked to clinical practice and service delivery. This interpretation is supported by aged care simplification studies, including the Medication Regimen Simplification Guide for Residential Aged Care and related Australian care-based research [16,36], as well as by HIV simplification studies and treatment switch trials conducted by hospital-based and industry-affiliated teams [37,38]. Meanwhile, institution-level productivity may also be influenced by organizational structures, affiliation patterns, and database indexing practices and, therefore, should be interpreted with caution. At the collaboration level, collaboration patterns were similarly concentrated. International links were most evident between North America and Europe, with additional connections involving East Asia and Oceania, whereas institutional and author networks were organized into several clusters. This pattern suggests that the field has been shaped by a limited number of active research groups and broader cross-regional and cross-institutional collaboration may be needed to expand the evidence base across different health systems and care settings.

In this study, keyword burst analysis was used to identify terms receiving rapidly increasing attention over specific periods, whereas the interpretation of broader research hot spots was based on their consistency with cocitation structure, keyword clustering, and thematic evolution. A notable feature of medication regimen simplification is its extension across multiple disease areas and care settings. The complementary analyses suggest that medication regimen simplification has developed from an initially focused literature centered largely on antiretroviral therapy, fixed-dose combinations, and treatment-related toxicity into a broader area that also encompasses chronic disease management, long-term care, and medication regimen complexity. Within this broader development, HIV remained the most consistent research direction, with persistent signals from clusters related to “2-drug regimen,” “NRTI-sparing regimens,” and “single-tablet regimen,” together with thematic evolution results including “antiretroviral therapy,” “HIV,” and “bictegravir.” This continuity is likely related to the long-term nature of HIV treatment and the need to reduce regimen burden while maintaining adherence and efficacy [39,40]. Beyond HIV, the field has expanded into other long-term treatment settings, particularly diabetes and cardiovascular disease [41-43], with increasing attention to “type 2 diabetes,” “heart failure,” “blood pressure control,” “basal insulin,” and “glucagon-like peptide-1 receptor agonists.” However, this expansion remains uneven across disease areas. HIV, diabetes, and selected cardiovascular topics were more prominently represented in the keyword and clustering analyses, whereas depression and Alzheimer disease were present in the keywords but did not form independent clusters or clearly defined thematic pathways. It may reflect variation in dominant research priorities across disease areas, with some fields historically placing greater emphasis on efficacy or survival-oriented outcomes rather than focusing on adherence or simplification [44,45]. It may also reflect the greater difficulty evaluating regimen simplification in mental health disorders and neurodegenerative diseases, where treatment and medication management are often individualized and shaped by cognitive impairment and caregiving involvement [46,47]. Further research should examine regimen simplification more explicitly in mental health, cognitive impairment, and multi-morbidity to clarify the indications, implementation, and long-term outcomes.

A central challenge in this field is no longer whether medication regimen simplification is feasible in principle but whether it can be implemented and sustained in routine care. Existing studies have focused mainly on populations with long-term medication use and high treatment burden, particularly older adults, people receiving community care, and those in long-term care settings [23,48,49]. In these populations, implementation depends not only on the regimen itself but also on cognitive and functional status, caregiver involvement, and the level of existing medication support [8,23,50]. Furthermore, simplification may also remain a clinical and pharmaceutical balancing process. Its real-world application requires consideration of effectiveness, safety, adherence, dosage form, dosing frequency, drug interactions, and pharmacokinetic constraints [8,13]. In this sense, regimen simplification is better understood as part of comprehensive medication management than as a simple act of discontinuing or switching treatment. Additionally, system-level factors may further shape implementation. In routine practice, medication simplification strategies may be limited by unclear workflows, insufficient multidisciplinary collaboration, and poor integration into everyday care processes [51,52]. These barriers may be more prominent in long-term care and community settings, where decision-making often depends on coordination among physicians, pharmacists, nurses, and caregivers [8]. These findings suggest that the future development of this field may depend not only on identifying effective simplification strategies but also on understanding how they can be embedded, coordinated, and sustained across real-world care settings.

The wider implementation of medication regimen simplification is likely to depend on policy and system conditions that support its integration into routine care. Reimbursement and reporting requirements may facilitate this process by providing institutional support for service delivery. For example, in the United States, Medicare Part D requires sponsors to establish medication therapy management programs and incorporate them into plan benefit structures and annual reporting requirements [53]. Quality measurement is another key element to routine implementation [54]. Although existing medication-related indicators can support accountability for medication use and safety [55,56], they seldom capture whether regimens have become simpler, whether simplification recommendations have been implemented, or whether treatment burden has been reduced. Therefore, more targeted indicators of regimen complexity, implementation, and treatment burden may be needed. Furthermore, implementation is also likely to depend on clearer professional roles and better workflow integration. In real-world practice, simplification is rarely a single prescription event but, rather, an ongoing process of assessment, decision-making, implementation, and follow-up. Clearer role allocation across physicians, pharmacists, and nurses may improve implementation [8,23,52]. In this context, the formalization of pharmacist prescribing pathways in Australia may support a more structured role for pharmacists in simplification-related care [57]. Additionally, digital support should be considered within the same implementation framework. Its value is likely to depend less on stand-alone tools than on the integration of complexity assessment, simplification prompts, and follow-up monitoring into existing workflows. Previous studies suggest that electronic decision support is more likely to be scalable when combined with routine structured medication review in long-term care [58,59]. Therefore, a more feasible approach may be to embed simplification-related functions within established care processes, including electronic health records, postdischarge medication reconciliation, and medication review in long-term care and chronic disease follow-up. Beyond reviews that focus on prescribing decisions or regimen optimization in specific patient groups [60,61], this study highlights a broader implementation-oriented perspective by linking bibliometric patterns to policy conditions for routine care.

### Limitations

Several limitations should be considered when interpreting the findings. First, this analysis was restricted to English-language publications indexed in the WoSCC. Therefore, relevant studies from other databases or in other languages may not have been captured, which could affect the completeness of the research landscape reported. Second, because medication regimen simplification is not a standardized indexing term, the search strategy relied on free-text terms and an operational definition, which may have resulted in the exclusion of conceptually relevant studies using different terminology. Third, bibliometric indicators describe patterns of publication, citation, and thematic structure, and recently published articles may have been underestimated because of citation lag. In addition, despite software-assisted standardization and manual checking, residual errors in author and institutional name disambiguation may have remained. Such errors can be reduced but are difficult to eliminate completely in bibliometric datasets, particularly for common surnames and institutions with multiple name variants, which could have affected the identification of prolific authors or institutions and the collaboration network analysis.

### Conclusions

This bibliometric analysis indicates a clear increase in publications on medication regimen simplification over the past 2 decades, particularly in recent years. Quantitative mapping further indicated that the literature has concentrated mainly on HIV, diabetes, and cardiovascular-related management, whereas some areas such as mental health and cognitive disorders remain comparatively underrepresented. Overall, the field is expanding, but its thematic development is still uneven. Future studies should further clarify the definition and scope of medication regimen simplification, pay more attention to underexplored diseases and care settings, and strengthen the evidence on implementation and patient-relevant outcomes. Cross-national and cross-system research will also be important for developing quality indicators, implementation pathways, and policy frameworks to support the wider use of medication regimen simplification in real-world practice.

## Supplementary material

10.2196/82274 Multimedia Appendix 1 Top 10 countries in terms of number of publications and citations.

10.2196/82274 Multimedia Appendix 2 Top 20 keywords in terms of frequency of occurrence and their corresponding centrality.

## Abbreviations

MeSH: Medical Subject Headings
WoSCC: Web of Science Core Collection
