# Supplementary material for: Global Trends and Research Hot Spots in Medication Regimen Simplification: Bibliometric Analysis
Source: JMIR Aging. 2026 May 29;9:e82274. doi: 10.2196/82274 (PMC13221124; doi:10.2196/82274)
Supplement: Multimedia Appendix 1 [file aging-v9-e82274-s001.docx]

**Supplementary Table 1** |Top 10 country in terms of number of publications and citations.

| **Rank** | **Country** | **Publications** | **Citations** |
| --- | --- | --- | --- |
| 1 | USA | 163 | 6720 |
| 2 | Italy | 75 | 3800 |
| 3 | UK | 61 | 3409 |
| 4 | Spain | 50 | 2034 |
| 5 | Australia | 41 | 2398 |
| 6 | France | 34 | 1517 |
| 7 | Germany | 30 | 1395 |
| 8 | Canada | 29 | 1436 |
| 9 | China | 26 | 418 |
| 10 | Belgium | 22 | 3028 |
