# Supplementary material for: Global Trends and Research Hot Spots in Medication Regimen Simplification: Bibliometric Analysis
Source: JMIR Aging. 2026 May 29;9:e82274. doi: 10.2196/82274 (PMC13221124; doi:10.2196/82274)
Supplement: Multimedia Appendix 2 [file aging-v9-e82274-s002.docx]

**Supplementary Table 2** | Top 20 keywords in terms of frequency of occurrence and the corresponding centrality

| **Rank** | **Keyword** | **Freq** | **Centrality** |
| --- | --- | --- | --- |
| 1 | efficacy | 63 | 0.13 |
| 2 | therapy | 61 | 0.18 |
| 3 | adherence | 61 | 0.19 |
| 4 | antiretroviral therapy | 53 | 0.16 |
| 5 | medication adherence | 52 | 0.09 |
| 6 | double blind | 46 | 0.26 |
| 7 | safety | 41 | 0.09 |
| 8 | management | 29 | 0.16 |
| 9 | open label | 27 | 0.07 |
| 10 | impact | 27 | 0.04 |
| 11 | risk | 25 | 0.09 |
| 12 | basal insulin | 25 | 0.05 |
| 13 | type 2 diabetes | 22 | 0.06 |
| 14 | nonadherence | 22 | 0.06 |
| 15 | care | 21 | 0.08 |
| 16 | adults | 20 | 0.13 |
| 17 | outcm | 19 | 0.01 |
| 18 | hiv infected patients | 19 | 0.07 |
| 19 | quality of life | 18 | 0.04 |
| 20 | dual therapy | 17 | 0.05 |
